# Supplementary material for: A foundation machine learning potential with polarizable long-range interactions for materials modelling
Source: Nat Commun. 2025 Nov 25;16:10484. doi: 10.1038/s41467-025-65496-3 (PMC12647596; doi:10.1038/s41467-025-65496-3)
Supplement: Supplementary file 1 — Supplementary Information [file 41467_2025_65496_MOESM1_ESM.pdf]

# Supplementary Information for

# A foundation machine learning potential with polarizable long-range interactions for materials modelling

Rongzhi Gao[1], ChiYung Yam[2,3], Jianjun Mao[2], Shuguang Chen[2,4], GuanHua Chen[1,2,*] & Ziyang Hu[1,2,*]

[1]Department of Chemistry, The University of Hong Kong, Pokfulam, Hong Kong SAR, China. [2]Hong Kong Quantum AI Lab Limited, Pak Shek Kok, Hong Kong SAR, China. [3]Shenzhen Institute for Advanced Study, University of Electronic Science and Technology of China, Shenzhen, China. [4]MattVerse Limited, Pak Shek Kok, Hong Kong SAR, China.

***Corresponding authors**: GuanHua Chen and Ziyang Hu

**Email:** ghc@everest.hku.hk or hzy@yangtze.hku.hk

# Table of contents

Supplementary Note 1. Polarizable charge equilibration formalism under external electric field ........ 3
Supplementary Note 2. Derivation of atomic forces ........ 5
Supplementary Note 3. Performance on dataset with different charge states and charge transfer ........ 6
Supplementary Note 4. Benchmarking on the foundation models ........ 8
Supplementary Note 5. Water molecules under external electric field ........ 10
Supplementary Note 6. Benchmarking on computational efficiency ........ 11
Supplementary Note 7. Transferability of our foundation model for long-range physics ........ 12
Supplementary Note 8. Transferability of our foundation model for polarization ........ 13
Supplementary Note 9. Finetuning for more refined potential energy surfaces ........ 15
Supplementary Note 10. Development of PQEq parameters ........ 17
Supplementary Note 11. Comparison between our bespoke models and non-charge-equilibration method models ........ 18
Supplementary Note 12. Polarization of $BaTiO_3$ ........ 19
Supplementary Note 13. Solid-electrolyte interphases of $Li_6PS_5Cl$/Li ........ 20
Supplementary Figures 1-13 ........ 21
Supplementary Tables 1-10 ........ 34
Supplementary References ........ 40

**Supplementary Note 1. Polarizable charge equilibration formalism under external electric field**

Polarizable charge equilibration (PQEq) [1-3] method assumes that each atom consists of a Gaussian charge core and shell. The shell charge ($q_{is}$) of atom $i$ has a fixed charge of $-z_i$, while the core charge ($q_{ic}$) is atomic partial charge ($q_i$) plus the fixed charge $z_i$. Here $z$ is set to 1 for all atoms [1]. The potential energy of PQEq is based on the second order expansion with respect to charge fluctuations,

$$\begin{aligned} E_{\mathrm{PQEq}} = & \sum_{i}^{N} \left( \chi_i^0 q_i + \frac{1}{2}\eta_i^0 q_i^2 + \frac{1}{2} K_s^i |\mathbf{r}_{ic} - \mathbf{r}_{is}|^2 \right) \\ & + \frac{1}{2}\sum_{i \neq j} \left( C(\mathbf{r}_{ic,jc}) q_{ic} q_{jc} + C(\mathbf{r}_{ic,js}) q_{ic} q_{js} + C(\mathbf{r}_{is,jc}) q_{is} q_{jc} + C(\mathbf{r}_{is,js}) q_{is} q_{js} \right). \end{aligned} \tag{1}$$

The electrostatic energy related term $C_{ik,jl}(\mathbf{r})$ between two Gaussian charges is given by,

$$C_{ik,jl}(\mathbf{r}) = \frac{1}{|\mathbf{r}|} \mathrm{erf}\left( \sqrt{\frac{\alpha_{ik}\alpha_{jl}}{\alpha_{ik} + \alpha_{jl}}} |\mathbf{r}| \right), \tag{2}$$

where $\alpha$ is the width of the Gaussian distribution [1]. With the constraint $\sum q_i = Q$ (net charge conservation condition) and the method of Lagrange multiplier ($E_\lambda = E_{\mathrm{PQEq}} - \lambda \sum q_i$), the linear equations are obtained by requiring $-\frac{\partial E_\lambda}{\partial q_i} = 0$,

$$\begin{bmatrix} H_{11} & H_{12} & \cdots & H_{1n} & -1 \\ H_{21} & H_{22} & \cdots & H_{2n} & -1 \\ \vdots & \vdots & \ddots & \vdots & \vdots \\ H_{n1} & H_{n2} & \cdots & H_{nn} & -1 \\ 1 & 1 & \cdots & 1 & 0 \end{bmatrix} \begin{bmatrix} q_1 \\ q_2 \\ \vdots \\ q_n \\ \lambda \end{bmatrix} = \begin{bmatrix} B_1 \\ B_2 \\ \vdots \\ B_n \\ Q \end{bmatrix}, \tag{3}$$

where

$$H_{ij} = \begin{cases} \eta_i^0, & i = j \\ C(\mathbf{r}_{ic,jc}), & i \neq j \end{cases}, \tag{4}$$

$$B_i = -\chi_i^0 - \sum_{j, i \neq j} \left[ C(\mathbf{r}_{ic,jc}) - C(\mathbf{r}_{ic,js}) \right] z_j. \tag{5}$$

By solving these linear equations, the partial charges can be obtained. Given the partial charges, new shell positions can be updated by the one-step Newton-Raphson method.

Generally, under an external electric field ($\boldsymbol{\epsilon}$), the shell positions are updated as following,

$$\mathbf{r}_{is,\,\mathrm{new}} = \mathbf{r}_{is,\,\mathrm{old}} + \frac{\mathbf{F}_{\mathrm{inter}} + \mathbf{F}_{\mathrm{external}} + \mathbf{F}_{\mathrm{intra}}}{E''\left(\mathbf{r}_{is,\mathrm{old}}\right)} \tag{6}$$

$$\mathbf{r}_{is,\,\mathrm{new}} \approx \mathbf{r}_{ic} + \frac{-\frac{\partial}{\partial \mathbf{r}_{is}}\left(\sum_j \left(C\left(\mathbf{r}_{is,jc}\right) q_{is} q_{jc} + C\left(\mathbf{r}_{is,js}\right) q_{is} q_{js}\right)\right)|_{\mathbf{r}_{is,\mathrm{old}}} + q_{is}\boldsymbol{\epsilon}}{K_s}. \tag{7}$$

Forces on the shell $\mathbf{F}_{\mathrm{inter}}$, $\mathbf{F}_{\mathrm{external}}$ and $\mathbf{F}_{\mathrm{intra}}$ originate from core-shell Coulombic interactions, external electric field, and the core-shell spring interactions, respectively. After the new shell positions are obtained, they can be used to update the partial charges until convergence. The partial charges and new shell positions will in return be used to compute the electrostatic potential with Supplementary Eq. 1. Following the Born-Oppenheimer adiabatic approximation, we consider the shell to be massless. For each molecular dynamics step, the shell is updated instantly. The forces on the core under external electric field can be expressed as,

$$\mathbf{F}_{ic,\mathrm{PQEq}} = -\frac{\partial E_{\mathrm{PQEq}}}{\partial \mathbf{r}_{ic}} + q_{ic} \cdot \boldsymbol{\epsilon}. \tag{8}$$

**Supplementary Note 2. Derivation of atomic forces**

The total forces of our model are,

$$\mathbf{F}_i = -\frac{\partial E_{\text{pot}}}{\partial \mathbf{r}_i} - \sum_j \frac{\partial E_{\text{pot}}}{\partial q_j}\frac{\partial q_j}{\partial \mathbf{r}_i}, \tag{9}$$

where $E_{\text{pot}}$, $\boldsymbol{r}_i$, and $q_i$ are total potential energy as described in Eq. 1 in the main text, atomic coordinates and partial charges, respectively. The Lagrange multiplier method ($E_\lambda = E_{\text{pot}} - \lambda \sum q_j$) requires,

$$-\frac{\partial E_\lambda}{\partial q_j} = 0, \tag{10}$$

so that,

$$\frac{\partial E_{\text{pot}}}{\partial q_j} = \lambda. \tag{11}$$

Thus, the forces are simplified as,

$$\mathbf{F}_i = -\frac{\partial E_{\text{pot}}}{\partial \mathbf{r}_i} - \lambda \sum_j \frac{\partial q_j}{\partial \mathbf{r}_i}. \tag{12}$$

And with the constraint,

$$\sum q_j = Q, \tag{13}$$

we have $\frac{\partial Q}{\partial r_i} = 0$ since the net charge $Q$ is a constant. Finally, the atomic forces are,

$$\mathbf{F}_i = -\frac{\partial E_{\text{pot}}}{\partial \mathbf{r}_i}. \tag{14}$$

Note that the gradient updates are stopped in the charge equilibration process to ensure partial derivatives are taken. For stresses, similar arguments hold. The virial stress (**S**) on the cell can be calculated via automatic differentiation of potential energy (Eq. 1) with respect to strain **s**,

$$\mathbf{S}_{ab} = \frac{1}{V_{\text{cell}}} \cdot \frac{\partial E_{\text{pot}}}{\partial \mathbf{s}_{ab}}. \tag{15}$$

where $V_{\text{cell}}$ is the volume of the cell.

## Supplementary Note 3. Performance on dataset with different charge states and charge transfer

The dataset compiled by Ko et al. [4] encompasses four distinct subsets, each characterized by specific charge configurations. These subsets include neutral, cationic, and anionic structures, where long-range electrostatic interactions play a crucial role in charge transfer. The dataset contains Ag clusters with positive and negative total charge, ($Ag_3^{+/-}$), Na-Cl ionic clusters with one neutral Na removed ($Na_{8/9}Cl_8^{+}$), hydrogenated carbon chains in both neutral and cationic states ($C_{10}H_2$/$C_{10}H_3^{+}$), and a periodic system consisting of Au clusters adsorbed on a MgO-(001) surface.

We trained models on these four datasets, and the test set performance was benchmarked against the fourth-generation high-dimensional neural network potential (4G-HDNNP) [4], with results presented in Table 1 in the main text. The PQEq parameters described in Supplementary Table 7 were employed for all systems except the Ag clusters, for which the parameters were re-optimized simultaneously during the training process. As shown in Table 1, our framework demonstrates superior performance in force prediction accuracy compared to 4G-HDNNP, despite exhibiting marginally higher energy prediction errors. Nevertheless, these energy deviations remain within acceptable bounds.

As shown in Supplementary Fig. 1 a, even not explicitly training on charge distributions, our model successfully captured the centrosymmetry of charges of the linear chain in $C_{10}H_2$. Although the absolute values of charges exhibit discrepancies, they did not compromise the accuracy of energy and force predictions. For the cationic carbon chains, the charge distributions assigned by PQEq closely align with density functional theory (DFT) calculations, demonstrating qualitatively consistent results across different charge partitioning schemes. For Na-Cl clusters, our model achieves comparable accuracy in reproducing DFT potential energy surfaces w.r.t. Na atom displacement as shown in Supplementary Fig. 1 b. For Ag clusters, where the energy depends on the cluster's overall charge state, the degeneracy between atomic structures and potential energy surfaces posed a significant challenge. The unmodified PQEq parameters are not adequate to model such strong charge-transfer systems. However, by concurrently optimizing the Ag parameters during the training process, we achieved low force errors with relative ease. Subsequent geometric optimization of Ag clusters using our model yielded structures that showed excellent agreement with DFT results as shown in Supplementary Fig. 1 c. The optimized Ag parameters are listed in Supplementary Table 8. For $Au_2$ adsorption on MgO surfaces, two primary adsorption geometries exist: a dimer in an upright "non-wetting" orientation anchored to surface oxygen, and a "wetting" configuration

parallel to the surface where two Au atoms reside above two Mg atoms. While the minimum energy structure on pristine MgO exhibits the “non-wetting” geometry, the flat “wetting” geometry becomes more energetically favourable when MgO is doped with three aluminium atoms. As demonstrated in Supplementary Table 1, we compare the wetting and non-wetting energy difference across different methods. Our model predictions demonstrate agreement with DFT calculations.

When comparing our PQEq partial charges with reference DFT Hirshfeld charges, we observe varying degrees of agreement across different chemical systems as shown in Supplementary Table 2. While some systems show remarkably agreement, particularly carbon-based systems and metal-oxide interfaces, it is important to acknowledge that direct numerical comparisons between different charge partitioning schemes have inherent limitations. The different partitioning philosophies mean that perfect agreement is neither expected nor necessarily desirable as a validation metric. As for training the different charge state dataset, the neural network architectures are provided in Supplementary Table 3. For feature multiplicity, we consistently employed 32 across all models to maintain all parameters in accordance with the reference [5].

**Supplementary Note 4. Benchmarking on the foundation models**

We performed a comprehensive comparison between models incorporating (our model) and excluding (w/o-lr model) polarizable long-range interactions. Both models were trained using identical machine learning potential frameworks and the MPtrj [6] dataset (detailed in Methods section of the main text). The training performance metrics are summarized in Supplementary Table 4. The results demonstrate that the incorporation of long-range interactions consistently enhances the accuracy of the model across all evaluation metrics.

Then, we conducted benchmarking of the bulk modulus using the w/o-lr model on the 10,154 materials sourced from Materials Project [7] same as described in the main text. Data points for which modulus calculations failed were excluded from the analysis. As shown in Supplementary Fig. 2, our findings demonstrate that our model incorporating polarizable long-range interactions consistently outperforms the model without long-range interactions.

Furthermore, we identified significant limitations in models lacking long-range interactions, particularly in their ability to accurately characterize layered materials. To illustrate this limitation, we selected lithium iron phosphate ($LiFePO_4$), a representative cathode material, as our model system. The simulation was performed on a supercell containing 672 atoms using molecular dynamics within the isothermal-isobaric ensemble, employing a 1 fs time step. The temperature protocol consisted of three sequential stages:

- Initial velocities generation at 5 K for 10 ps
- Temperature ramping from 5 K to 1000 K over 50 ps
- Extended equilibration at 1000 K for 500 ps.

Previous experimental [8] and theoretical studies [9] have established that $LiFePO_4$ maintains a stable olivine structure at 1000 K without phase transitions. The simulation results, presented in Supplementary Fig. 3, reveal that the w/o-lr model predicts an unrealistic irreversible phase transition at 1000 K. This unrealistic transformation likely stems from the model's inadequate representation of interlayer interactions. Conversely, our model successfully reproduces this experimentally observed thermal stability, validating its accuracy in describing the structural dynamics of layered materials.

We also conducted benchmarking with CHGNet [6] and MACE-MP-0 (large version) [10] on the test sets provided by the reference [11]. For near-equilibrium evaluations, two randomly sampled collections are used: structures from the MPtrj dataset [6] (MPtrj-random) and from Alexandria dataset [12] (Alexandria-random). These assess model accuracy on energetically favourable configurations, which is crucial for predicting material stability. Three increasingly

challenging thermodynamic perturbation (TP) test sets evaluate performance under varied temperature and pressure conditions with non-equilibrium atomic configurations. MPF-Alkali-TP focuses on potential ionic conductors, comprising 50 randomly selected compounds containing at least one alkali metal paired with electronegative elements (N, O, P, S, Se, or halogens) from MPF2021 dataset [13]. This dataset specifically tests model accuracy for materials with significant ionic character. MPF-TP broadens the scope with 50 randomly selected compounds from the MPF2021 dataset without elemental restrictions, presenting a more diverse chemical challenge. Random-TP represents the most demanding test, constructed by arbitrarily positioning 20 atoms of random elements within simulation boxes, creating hypothetical structures that test extreme generalization capability. All configurations including the Yb element were excluded. Supplementary Table 5 demonstrates the performance of our model across diverse test sets. While MACE-MP-0 performs well on the near-equilibrium dataset, it shows poor performance on the challenging TP dataset. In contrast, our model demonstrates good robustness on both the near-equilibrium and TP datasets. With respect to forces and stresses, which are the crucial quantities of interest for molecular dynamics, our model nearly outperforms both CHGNet and MACE-MP-0 on the TP test sets. It is important to note that our comparison was limited to universal models trained exclusively on zero-temperature dataset (MPtrj) and did not include MatterSim [11], which incorporates high-temperature datasets. The mean absolute errors (MAE) for the prediction ($\mathbf{x}^{\mathrm{pred}}$) and reference ($\mathbf{x}^{\mathrm{ref}}$) values are defined as,

$$\mathrm{MAE}\left(\mathbf{x}^{\mathrm{ref}}, \mathbf{x}^{\mathrm{pred}}\right) = \sum_{i}^{N} \frac{1}{N} \left| x_i^{\mathrm{ref}} - x_i^{\mathrm{pred}} \right|, \tag{16}$$

where for forces and stresses, the metric is calculated on a per-component basis.

## Supplementary Note 5. Water molecules under external electric field

In the polarization of static water molecules under an electrostatic field shown in Fig. 2b, the charge equilibration (QEq) model demonstrates limitations in handling intramolecular polarization when electric field strength varies in the *x*-axis. The QEq model represents atoms as either point charges or Gaussian charges, which restricts its ability to accurately capture molecular polarization effects. In contrast, the PQEq model separates atomic cores and shells (Supplementary Eq. 6), enabling it to effectively account for intramolecular polarization phenomena. To illustrate this difference, we present in Supplementary Fig. 4 the variations in O atom partial charge differences across different models as a function of the applied electric field strengths. It is important to note that our analysis does not directly compare the absolute values of partial charges, but rather employs relative values as a reference point. Under different charge partitioning schemes, comparing absolute values lacks meaningful interpretation, whereas the relative changes more accurately reflect how various methods respond to the applied electrostatic fields. Supplementary Fig. 4 demonstrates that our model shows consistency with first principle calculations in terms of charge variations. This comparison validates that our approach accurately captures the electronic response behaviour under various conditions.

We also examine the limitations of the QEq method when applied to water molecules under an external electric field, particularly its tendency to predict physically unrealistic charge distributions. To illustrate the distinctions between QEq and PQEq charge distribution methods in the presence of an electric field, we performed a simple demonstration following the protocol outlined in the reference [14]. Supplementary Fig. 5 a presents visualizations of water molecules using both QEq and PQEq methods. Supplementary Fig. 5 b and c depict the temporal evolution of corresponding *z*-positions and molecular charges within the simulation box for both methods at an electric field of 0.25 $V \cdot Å^{-1}$. Under QEq, the water molecules accumulate non-zero net charges and migrate to the top and bottom of the box [14]. In contrast, using our model, the water molecules maintain charge neutrality and remain stationary despite the applied electric field. This behaviour aligns with DFT calculations, which similarly predict no molecular displacement for the two water molecules.

## Supplementary Note 6. Benchmarking on computational efficiency

Our model introduces explicit polarizable long-range interactions while still maintaining good computational efficiency. To highlight the performance of this model, benchmarking was done on molecular dynamics simulations. Supercell models were generated from $Li_2PO_2N$ (mp-1020019) from Materials Project [7] and simulations were conducted. Additionally, the PyTorch-based universal machine learning interatomic potentials CHGNet [6] was used for comparison. All molecular dynamics simulations were carried out under the canonical ensemble simulations with a Nosé-Hoover thermostat [15, 16] at 300 K temperature. All the computations were performed with a single NVIDIA H100 GPU.

As shown in Supplementary Fig. 6, we present efficiency in terms of time consumption per simulation step. Although our model demonstrates $O(N^3)$ scaling due to inversion the Supplementary Eq. 3, it successfully handles large-scale systems exceeding 20,000 atoms, highlighting its applicability to complex materials simulations. In our studied reaction systems containing tens of thousands of atoms, the inversion method provides acceptable computational efficiency. Nevertheless, in future work, we plan to further refine our algorithms, such as implementing iterative solution methods to reduce complexity, thereby achieving more computationally efficient implementations and lowering resource consumption.

**Supplementary Note 7. Transferability of our foundation model for long-range physics**

In comparison with existing universal models, our model demonstrates transferability in describing dimer interactions. As mentioned in the main text, regarding the potential energy surface capturing of Na-Cl and Na-Na dimers, we have discovered that our model not only accurately reproduces the long-range behaviour predicted by DFT/PBE [17] calculations, but also effectively captures the potential energy surfaces of these dimers in the short-range region compared to CHGNet and MACE-MP-0, as illustrated in Supplementary Fig. 7. The enhanced transferability of our model across different dimers suggests its potential utility for a broad spectrum of applications requiring accurate representation of interatomic forces across multiple distance scales.

Another example that showcases the transferability of our model is its ability to distinguish between different charge states and accurately simulate their corresponding long-range interactions. We selected the OH-OH system as a case study, investigating the potential energy curves when the system consists of two neutral hydroxyl radicals versus when it carries a single negative charge. As illustrated in Supplementary Fig. 8, the plot compares the performance of our model against DFT (the ground truth), MACE-MP-0, and CHGNet for the system in both "ionic" and "neutral" states as a function of distance. For the neutral state (dashed lines), which represents the physical scenario of a weak, non-bonded interaction, the DFT reference shows a very shallow potential well with relative energies. Our model demonstrates good agreement with the DFT reference, accurately capturing the subtle attractive interaction. In contrast, MACE-MP-0 and CHGNet struggle to capture this long-range interaction; they appear less transferable than our model as they fail to reproduce the precise shape and depth of the DFT curve. For the ionic state (solid lines), which represents a strong, long-range Coulombic repulsion between charged species, the DFT reference shows a significant positive (repulsive) energy that decays slowly with distance. Our model correctly identifies the ionic state and qualitatively reproduces the long-range repulsive energy curve from DFT. Despite a slight overestimation of the energy, the model captures the physical trend and magnitude of the interaction across the entire distance range. Conversely, MACE-MP-0 and CHGNet are completely unable to distinguish the ionic from the neutral state. This represents a fundamental physical problem, indicating that their underlying architectures cannot capture charge-state-dependent long-range physics.

**Supplementary Note 8. Transferability of our foundation model for polarization**

A crucial test of a model's physical fidelity is its response to external fields. We evaluated our model's ability to predict the energy response of a system to a uniform electric field. We adopted a periodic system containing 64 water molecules with each cell length measured 12.4185 Å, as described in the reference [18]. All the water molecules are within *yz*-plane. The first and third layers are same while the second and fourth are same. An electric field was then applied along the *x*-axis with varying strength.

The results are presented in the Supplementary Fig. 10, which plots the system energy as a function of field strength. Our model demonstrates outstanding agreement with the DFT reference, accurately reproducing the energy curve across the entire field range from −2 to 2 V·Å$^{-1}$. Notably, the performance of our foundation model is on par with that of FIREANN [18], a state-of-the-art model that was specifically trained on this water system. This highlights the transferability of our model, as it correctly captures complex polarization physics without explicit training for this task. In contrast, the CHGNet and MACE-MP-0 models are entirely unresponsive to the field, predicting a constant energy. This represents a fundamental failure to capture one of the most essential physical interactions, underscoring the superior predictive power and physical realism of our model.

Conventional universal machine learning potentials like MACE-MP-0 and CHGNet are typically trained on field-free energies and forces, and their architectures lack the components to describe fundamental electrostatic properties like the dipole moment. Consequently, they are inherently incapable of predicting response properties such as polarizability, which is defined by the change in energy or dipole moment under an applied field. We further validated the powerful transferability of our model by assessing its ability to predict molecular polarizability, a key electronic response property. To accomplish this, we curated a challenging test set comprising 7,211 diverse small molecules from the QM-7b dataset [19-22]. For each molecule, the ground-truth isotropic polarizability ($\alpha_{\mathrm{iso}}$) was computed using a finite difference method,

$$\boldsymbol{\alpha} = \frac{\partial \boldsymbol{\mu}}{\partial \boldsymbol{\epsilon}} \tag{17}$$

$$\alpha_{\mathrm{iso}} = \frac{1}{3}\left(\alpha_{xx} + \alpha_{yy} + \alpha_{zz}\right) \tag{18}$$

where $\boldsymbol{\mu}$ and $\boldsymbol{\epsilon}$ are the dipole moment and electric field [23].

This involved calculating the system's energy response to a small, externally applied electric field—a physical process the model was not explicitly trained to reproduce. The results of this

evaluation are presented in the Supplementary Fig. 11. The plot, which compares our model's predictions against the reference values, demonstrates good agreement. The model achieves a MAE of just 4.57 atomic units (a.u.) across this vast and chemically diverse dataset. This high level of accuracy, achieved without direct training on polarizability data, provides compelling evidence for the model's strong generalization capabilities and its ability to capture the fundamental physics governing molecular electronic response. It is compelling evidence that our model has learned a transferable representation of the underlying electronic structure and its response to external stimuli—a capability that lies beyond the scope of other universal machine learning potential models.

**Supplementary Note 9. Finetuning for more refined potential energy surfaces**

While our model provides a foundational understanding, achieving *ab initio* accuracy for specific and challenging systems like charged clusters necessitates a refinement of the potential energy surface. To rigorously assess the physical fidelity of the foundation models, we conducted an evaluation on the potential energy surfaces (PES) of charged sodium chloride clusters from the dataset proposed by Ko et al. [4]. The initial test involved scanning the PES of the $Na_{8/9}Cl_8^+$ cluster systems along a coordinate defined by varying the distance between two sodium atoms. As illustrated in Supplementary Fig. 12 a, c, and e, a comparison with DFT calculations reveals that all three foundation models fail to accurately describe the PES for these systems. Notably, our model demonstrated a potential to distinguish between the two clusters due to the charge state descriptor, whereas other models did not.

We acknowledge that capturing such a refined PES requires model specialization, so we performed a finetuning procedure on all three models. A subset was created by randomly selecting 20% of the total 5000 configurations from the $Na_{8/9}Cl_8^+$ dataset. Regarding our finetuning strategy, we adopted a full finetuning approach. For our model and CHGNet, only the final two convolutional layers, and the multilayer perceptron were updated. For MACE-MP-0, the entire model was subjected to finetuning, as implemented in its own code. The finetuning protocol was as follows: the subset was partitioned into training and validation sets with a 4:1 ratio. The optimization objective was exclusively the atomic forces, using the Mean Squared Error (MSE) as the loss function. All models were trained for 1000 epochs with a batch size of 10 and a learning rate of $5\times10^{-4}$.

After finetuning, PES of our model for $Na_{8/9}Cl_8^+$ clusters achieve a good agreement with the DFT reference as shown in Supplementary Fig. 12 b. This demonstrates that by accurately learning the forces, the model implicitly reconstructs a highly precise and physically consistent potential energy surface, capturing the subtle energetic details that were absent in the foundation model. On the other hand, following the finetuning procedure, both CHGNet and MACE-MP-0 exhibited a reduction in error as shown in Supplementary Fig. 12 d and f. However, their final predicted potential energy surfaces settled into an intermediate position, averaging the two distinct DFT reference surfaces for two clusters. This indicates that while finetuning enables these foundation models to learn a more refined potential energy surface to some extent, they remain unable to differentiate between the two distinct clusters. We may attribute this limitation to the absence of explicit descriptors for the system's net charge within

their architectures, which prevents them from tweaking the unique energetic signature of each cluster.

Having established the efficacy of finetuning for isolated clusters, we extended our investigation to more complex periodic systems to assess the models' ability to capture subtle surface chemistry. We focused on the refined potential energy surface of an $Au_2$ dimer adsorbed on both Al-doped and undoped MgO surfaces, using the dataset provided by Ko et al. [4]. We found that message-passing-based foundation models, after finetuning, are indeed capable of learning these precise potential energy surfaces. The finetuning protocol was consistent with our previous tests: a subset comprising 20% of the configurations was randomly selected and partitioned into a 4:1 training-to-validation ratio. The models were then trained for 1000 epochs with a $5\times10^{-4}$ learning rate, exclusively minimizing the MSE loss on atomic forces. As summarized in Supplementary Table 6, both finetuned CHGNet and our model successfully reproduce the DFT equilibrium bond lengths on both doped and undoped structures. Nonetheless, our model achieves slightly higher fidelity with the DFT reference. In contrast, the inferior performance of MACE-MP-0 may be attributed to its limited effective receptive field arising from only two message-passing layers, which is likely insufficient to model the complex interactions modulated by the dopant.

In summary, our work establishes a powerful two-step approach: first, pretraining on a universal dataset to build a robust foundation model, and second, performing targeted, data-efficient finetuning to achieve ab initio accuracy for specific applications. The success of this second step is, however, contingent on the inherent capabilities of the model's architecture. A successful foundation model must not only learn general physics but also possess the necessary descriptors and structural flexibility—such as handling net charge and capturing long-range effects—to be effectively specialized for the diverse and challenging problems in modern materials science.

**Supplementary Note 10. Development of PQEq parameters**

The optimization of PQEq parameters aims at reproducing the Quantum Mechanics (QM) interaction energy. Due to inappropriate lithium parameters such as its small spring constant, the partial charges and shell position shifts of lithium atoms tend to be weird. We adopt the reference paper's method [3] to optimize the parameters of Li. Here, the genetic algorithm (GA) is used. The PQEq0 [1] parameters of H and Na are selected as the upper and lower bound. 50 population and 0.05 mutation probability are chosen as the hyper-parameters of the GA process. After optimization, the radius of Li is about 0.69 Å, which is close to its ionic radius of 0.76 Å [24]. The final optimized parameters for the foundation model are listed in Supplementary Table 7.

**Supplementary Note 11. Comparison between our bespoke models and non-charge-equilibration method models**

We also benchmarked our bespoke models against the non-charge-equilibration method models developed by Kim et al. (referred to as cace-lr) [25] on Ko et al. datasets [4]. For the $Ag_3^{+/-}$ and $Au_2$-MgO systems, the PQEq parameters for Ag and Au were specifically reoptimized to more accurately capture the long-range interactions. All other PQEq parameters were kept identical to those of the foundation model, as detailed in Supplementary Table 7. The results of this direct comparison, summarized in Supplementary Table 9, demonstrate that our methodology is highly competitive with this reference model, even when using geometry-invariant PQEq parameters.

**Supplementary Note 12. Polarization of $BaTiO_3$**

In a Ti-centred unit cell $BaTiO_3$, Ba atoms occupy the eight vertices of the hexahedron while O atoms occupy the face-centred site. Accordingly, the local polarization of each $BaTiO_3$ unit cell is defined as,

$$\mathbf{P} = \frac{1}{V}\left(\frac{1}{8}Z_{\mathrm{Ba}}^{*} \cdot \sum_{i=1}^{8} \mathbf{r}_{\mathrm{Ba},i} + Z_{\mathrm{Ti}}^{*} \cdot \mathbf{r}_{\mathrm{Ti},i} + \frac{1}{2}Z_{\mathrm{O}}^{*} \cdot \sum_{i=1}^{6} \mathbf{r}_{\mathrm{O},i}\right). \tag{19}$$

$V$ is the volume of the unit cell. $Z_{\mathrm{Ba}}^{*}$, $Z_{\mathrm{Ti}}^{*}$ and $Z_{\mathrm{O}}^{*}$ are the Born effective charges [26] of Ba, Ti, and O, respectively. $\mathbf{r}_{\mathrm{O},i}$, $\mathbf{r}_{\mathrm{Ti},i}$and $\mathbf{r}_{\mathrm{O},i}$ are the atomic coordinates.

**Supplementary Note 13. Solid-electrolyte interphases of $Li_6PS_5Cl$/Li**

The interface of the $Li_6PS_5Cl$/Li, which consists of 13,284 atoms, has been meticulously constructed with a dimension of 28.55 nm × 2.97 nm × 2.97 nm. The structure of $Li_6PS_5Cl$ is derived from the reference [27]. The lithium metal anode spans roughly 10 nm, and the electrolyte section extends nearly 18.5 nm in length. Supplementary Fig. 13 presents the initial and final structures obtained through 2-ns isothermal–isobaric molecular dynamics at 1 atm and 300 K, 400 K, and 500 K, respectively.

## Supplementary Figures 1-13

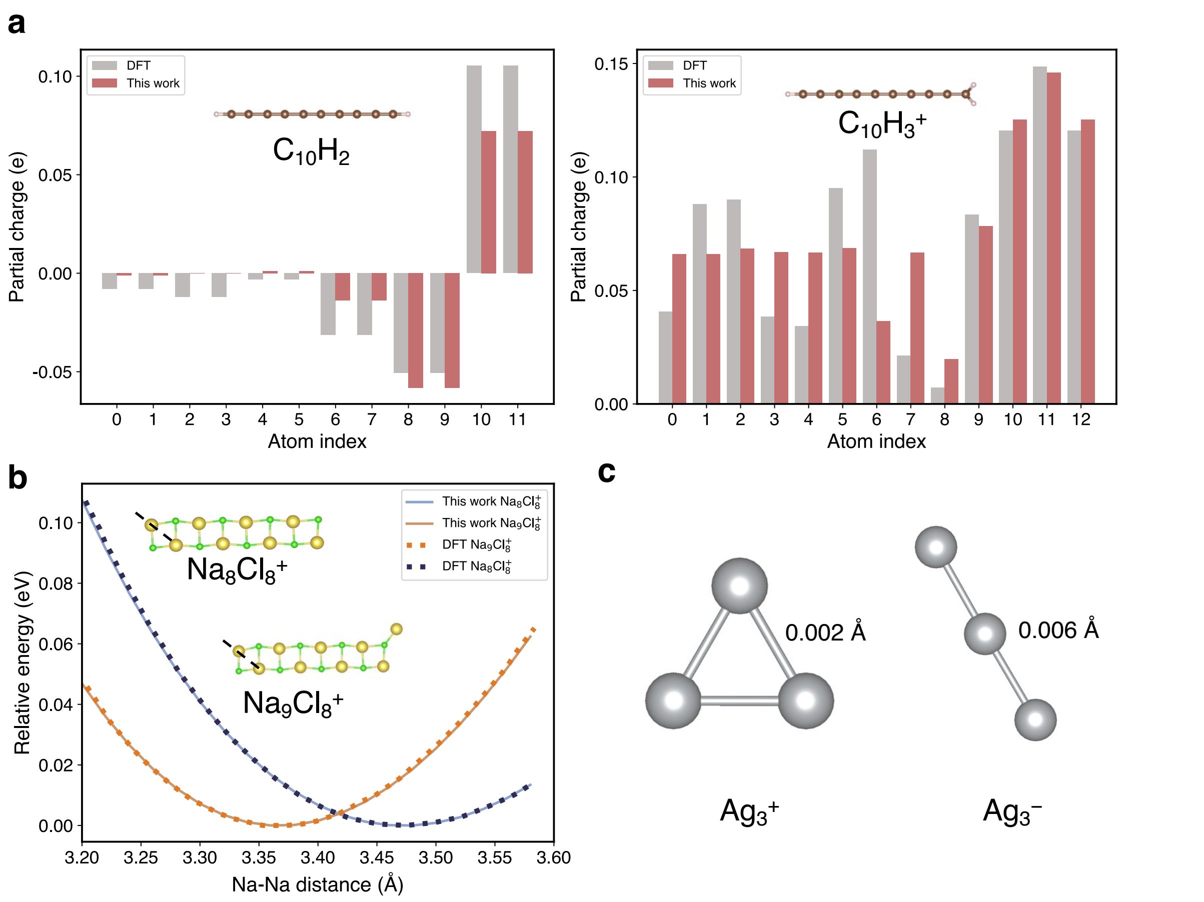


**Supplementary Fig. 1: Bespoke models' performance on different charge state dataset. a** Comparative analysis of atomic partial charges in $C_{10}H_2$ and $C_{10}H_3^+$ carbon chain molecules, depicted with brown carbon atoms and white hydrogen atoms. The analysis contrasts Hirshfeld charges [28] derived from density functional theory (DFT) calculations with the predictions of our model. **b** Potential energy surfaces of the $Na_{8/9}Cl_8^+$ cluster system, comparing DFT results with our model predictions. The surfaces were generated by varying the position of the first Na atom relative to the second Na atom as shown in dashed lines, with Na and Cl atoms represented in yellow and green, respectively. **c** Geometric optimization results for Ag clusters using our model, with root mean squared displacement (RMSD) values (in Å) calculated relative to DFT-optimized minima. All the DFT data are extracted from the reference [1].

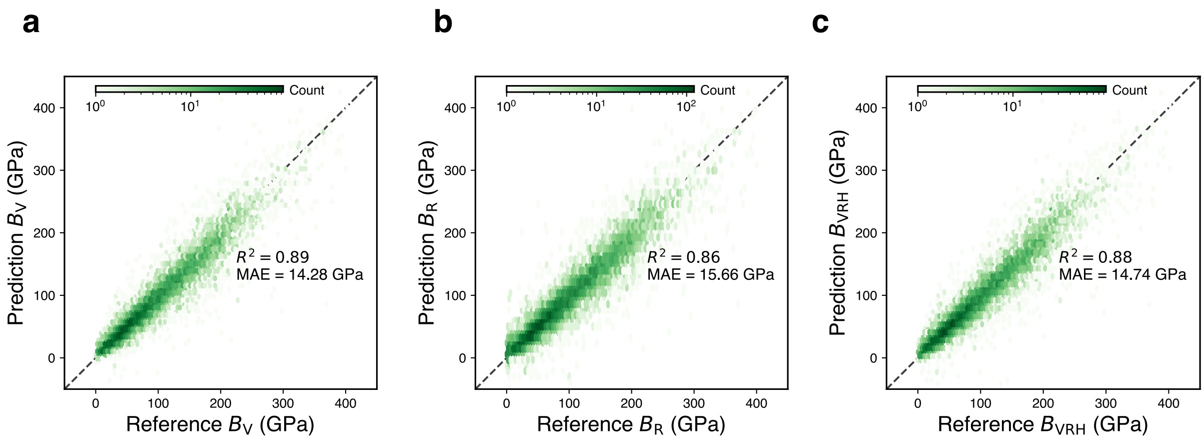


**Supplementary Fig. 2: Bulk modulus predictions of w/o-lr model.** Comparison of bulk modulus from reference density functional theory (DFT) calculations and the model without polarizable long-range interactions (w/o-lr model) predictions: **a** Voigt approach ($B_V$), **b** Reuss method ($B_R$), and **c** Hill average ($B_{VRH}$). The R-squared values ($R^2$) and mean absolute errors (MAE) of the bulk modulus are also shown.

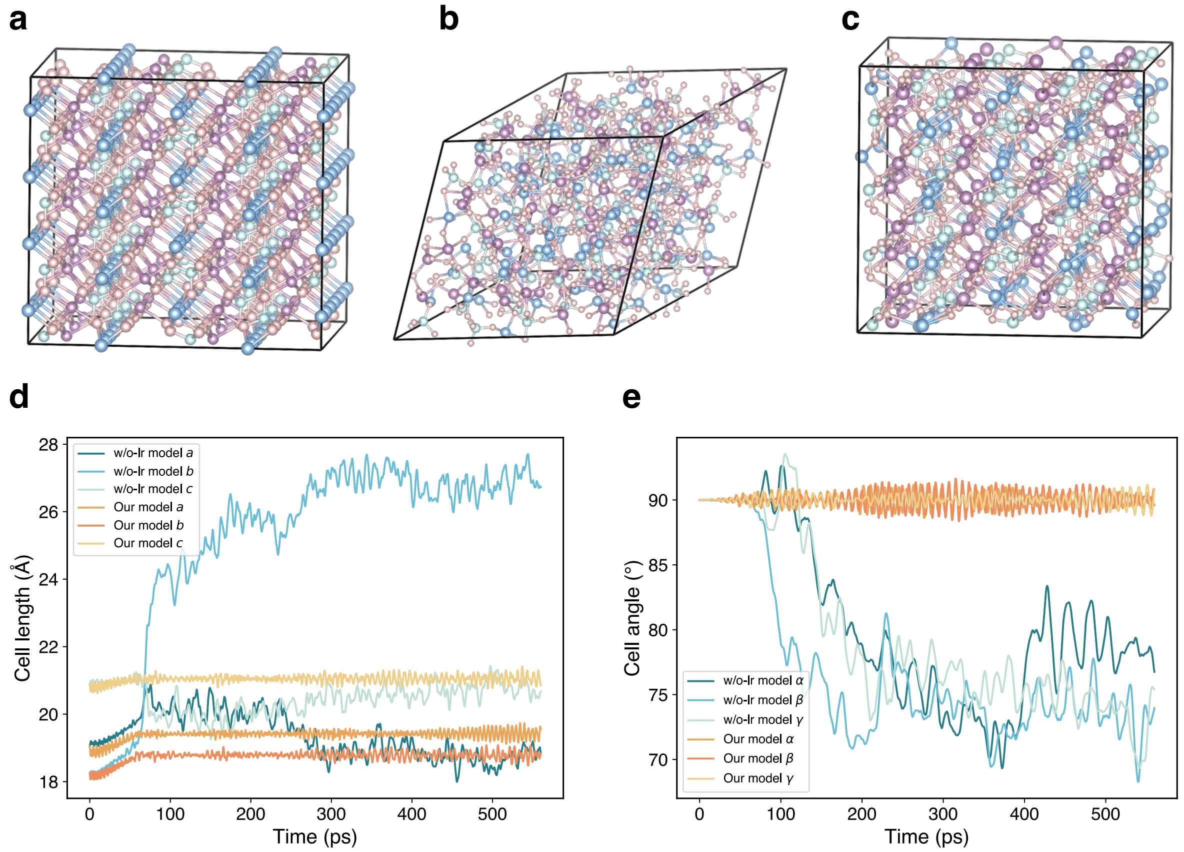


**Supplementary Fig. 3: Molecular dynamics simulations stability.** The molecular dynamics simulations are conducted on a typical cathode lithium iron phosphate ($LiFePO_4$) material. **a** the initial structure of $LiFePO_4$. Final configurations after 560 ps isothermal-isobaric ensemble simulations: **b** our model and **c** the model without polarizable long-range interactions (w/o-lr model). Lithium, iron, oxygen, and phosphorus atoms are depicted as blue, purple, light red and light green spheres. Evolution of lattice parameters **d** $a$, $b$, and $c$ **e** $\alpha$, $\beta$, and $\gamma$ during the molecular dynamic simulations.

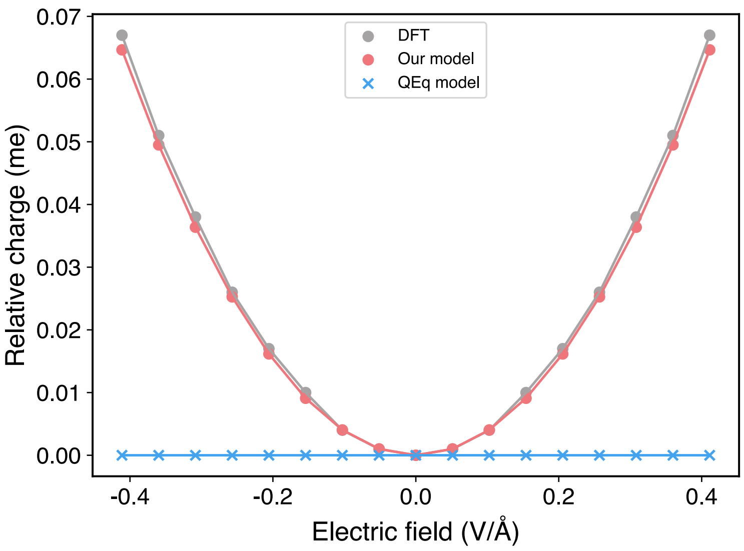


**Supplementary Fig. 4: Partial charges differences when applying different electric fields.** The variations in O atom relative partial charge differences between density functional theory (DFT) Hirshfeld calculations [28] (gray), our model (red), and the conventional charge equilibration (QEq) model (blue).

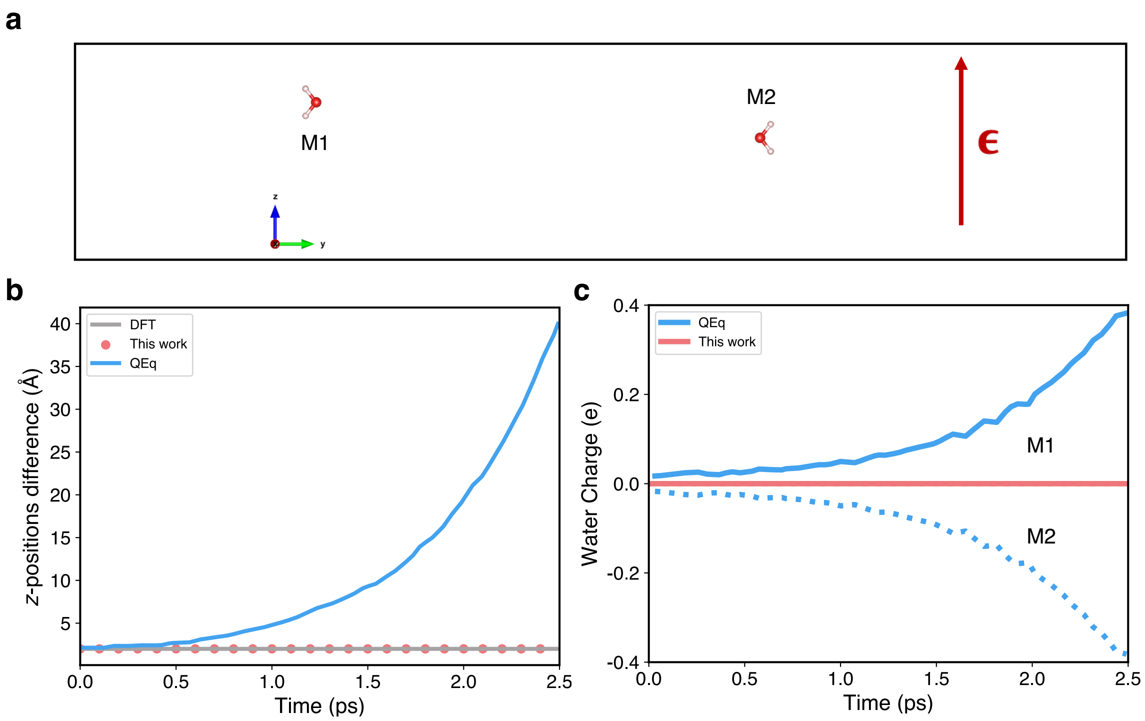


**Supplementary Fig. 5: Water molecules behaviours in dynamic condition. a** Visualization of two interacting water molecules (M1 and M2) under an applied electric field of 0.25 V·Å$^{-1}$ oriented along the positive $z$-axis. The water molecules model oriented in the $yz$-plane, with oxygen and hydrogen atoms shown in red and white, respectively. **b** Temporal evolution of $z$-positions difference ($|R_{\mathrm{M1},z} - R_{\mathrm{M2},z}|$) of the two water molecules and **c** charge dynamics of both water molecules calculated using charge equilibration (QEq) method (blue curves), our foundation model (red dots and curves), and density functional theory (DFT) reference (gray curves). Solid and dash lines represent two molecules, respectively. The QEq and DFT results are extracted from the reference [8].

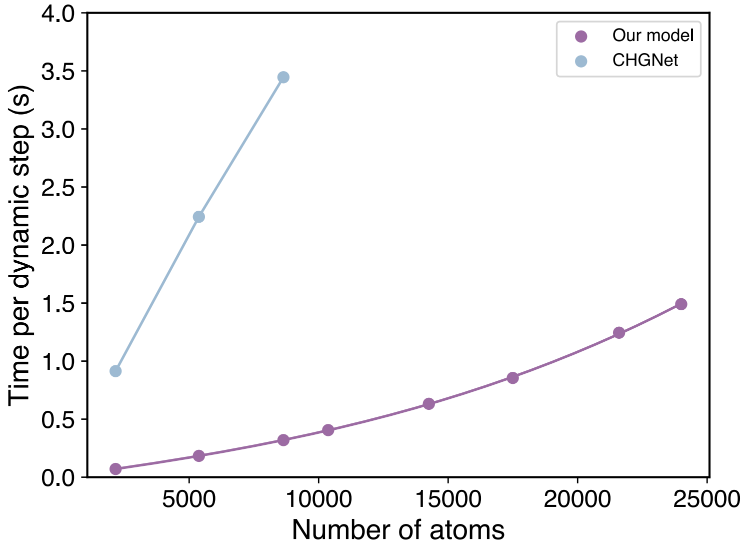


**Supplementary Fig. 6: Computational cost of our model.** Benchmarking on the computational efficiency involved in molecular dynamics simulations and geometry optimizations. All the calculations are performed on a single NVIDIA H100 GPU. The time consumption per step during 100 steps of molecular dynamics simulations for our model is presented in purple dots. Results for CHGNet [6] are shown in blue dots; however, the calculation for systems with more than 10 thousand atoms was halted due to out of GPU memory in the calculation environment.

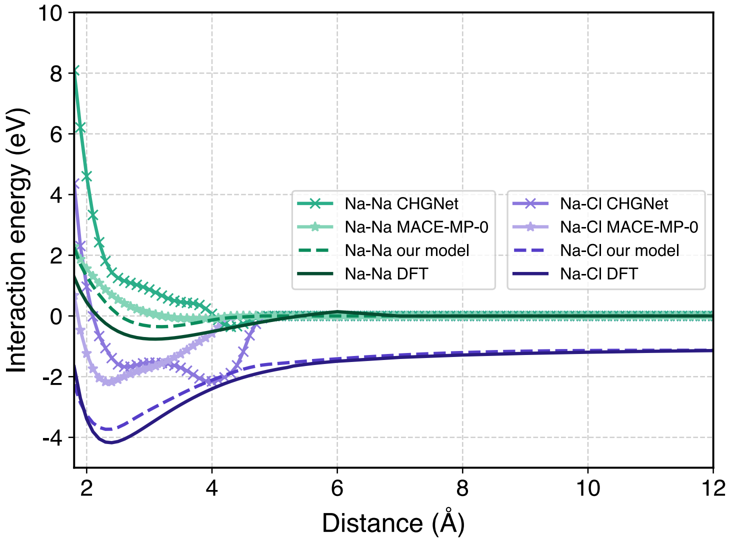


**Supplementary Fig. 7: Na-Na and Na-Cl dimers interaction energies.** The interaction energies for Na-Na (green) and Na-Cl (purple) dimers predicted by our model, CHGNet [6], MACE-MP-0 (large version) [10], and reference density functional theory (DFT) calculations.

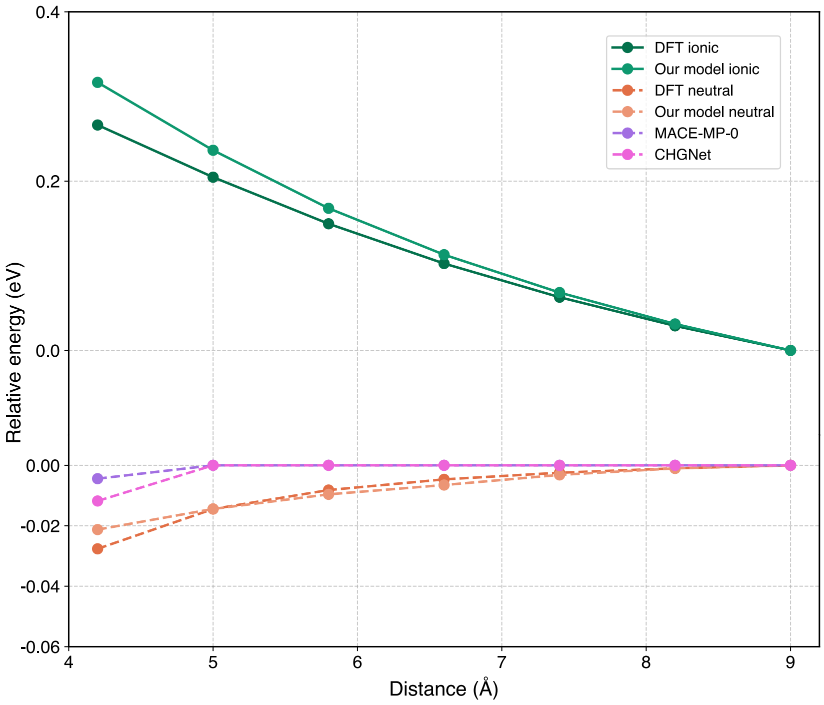


**Supplementary Fig. 8: Neutral and ionic state OH–OH systems relative energies.** Relative energy versus distance for an OH–OH system in its neutral (dashed lines) and ionic (solid lines) states. Our model's predictions are compared with density functional theory (DFT), MACE-MP-0 (in purple) [10], and CHGNet (in pink) [6]. The ionic state of our model is in light green and DFT is in dark green, while the neutral state of our model is in light orange and DFT is in dark orange, respectively. The results demonstrate that our model accurately reproduces the DFT reference for both the weak interaction of the neutral state and, crucially, the long-range Coulombic repulsion of the ionic state. Foundation models like MACE-MP-0 and CHGNet fail to capture the long-range physics of the ionic state.

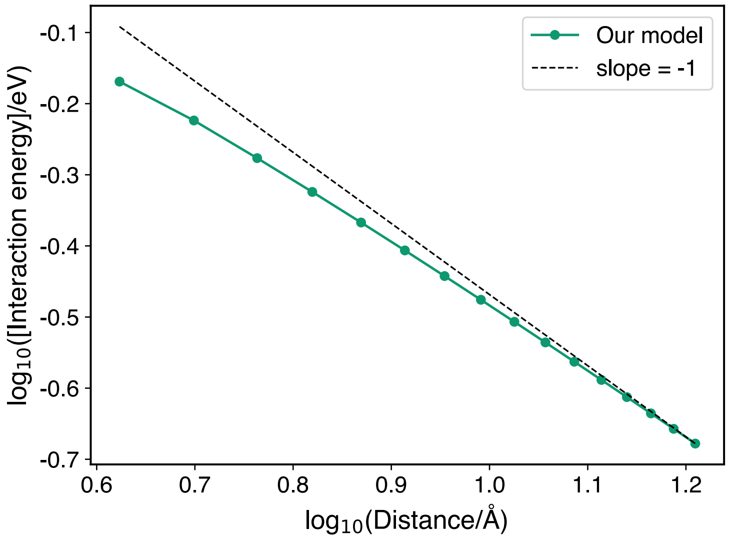


**Supplementary Fig. 9: Log-log plot of ionic state OH–OH dimer interaction energies of our model.** A log-log plot showing the interaction energy as a function of distance for an OH–OH dimer in an ionic state. The data from our model (solid green line) is compared with a reference line representing a pure $1/r$ decay (dashed black line).

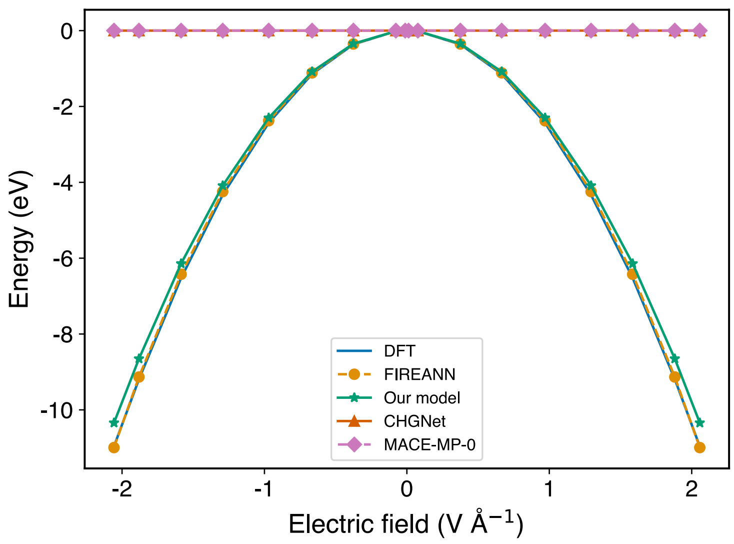


**Supplementary Fig. 10: Evaluation of polarizable interactions in the periodic water model.** The plot compares the energy and electric field curve of our model (in green) against density functional theory (DFT, in blue), FIREANN (in orange) [18], CHGNet (in red) [6], and MACE-MP-0 (in pink) [10].

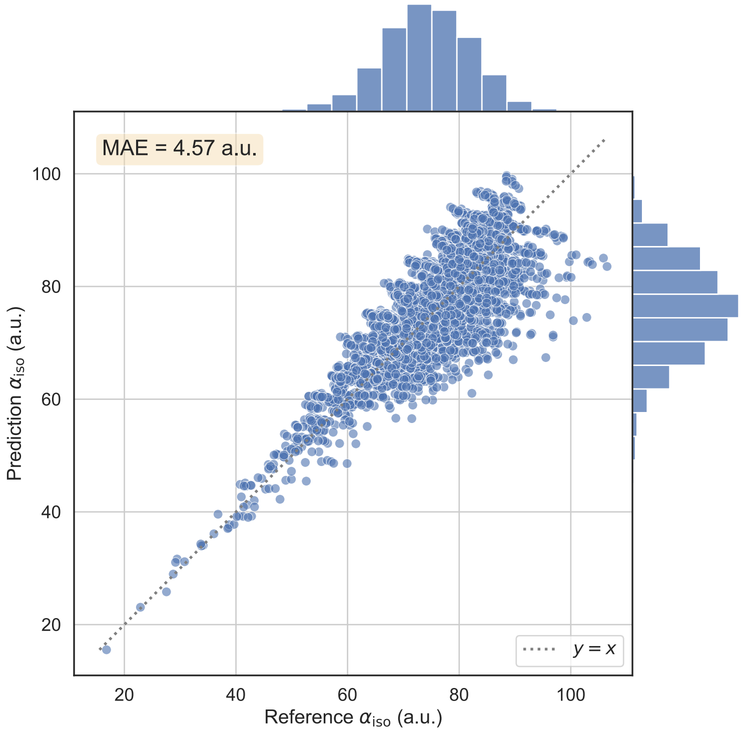


**Supplementary Fig. 11: Transferability of our model in molecular polarizability predictions.** Comparison of the predicted isotropic polarizability ($\alpha_{\text{iso}}$) against reference coupled-cluster theory including single and double excitations values for 7,211 molecules from the QM-7b dataset [19]. The model achieves a mean absolute error (MAE) of 4.57 atomic units (a.u.), demonstrating its strong capability to generalize and predict complex electronic response properties accurately.

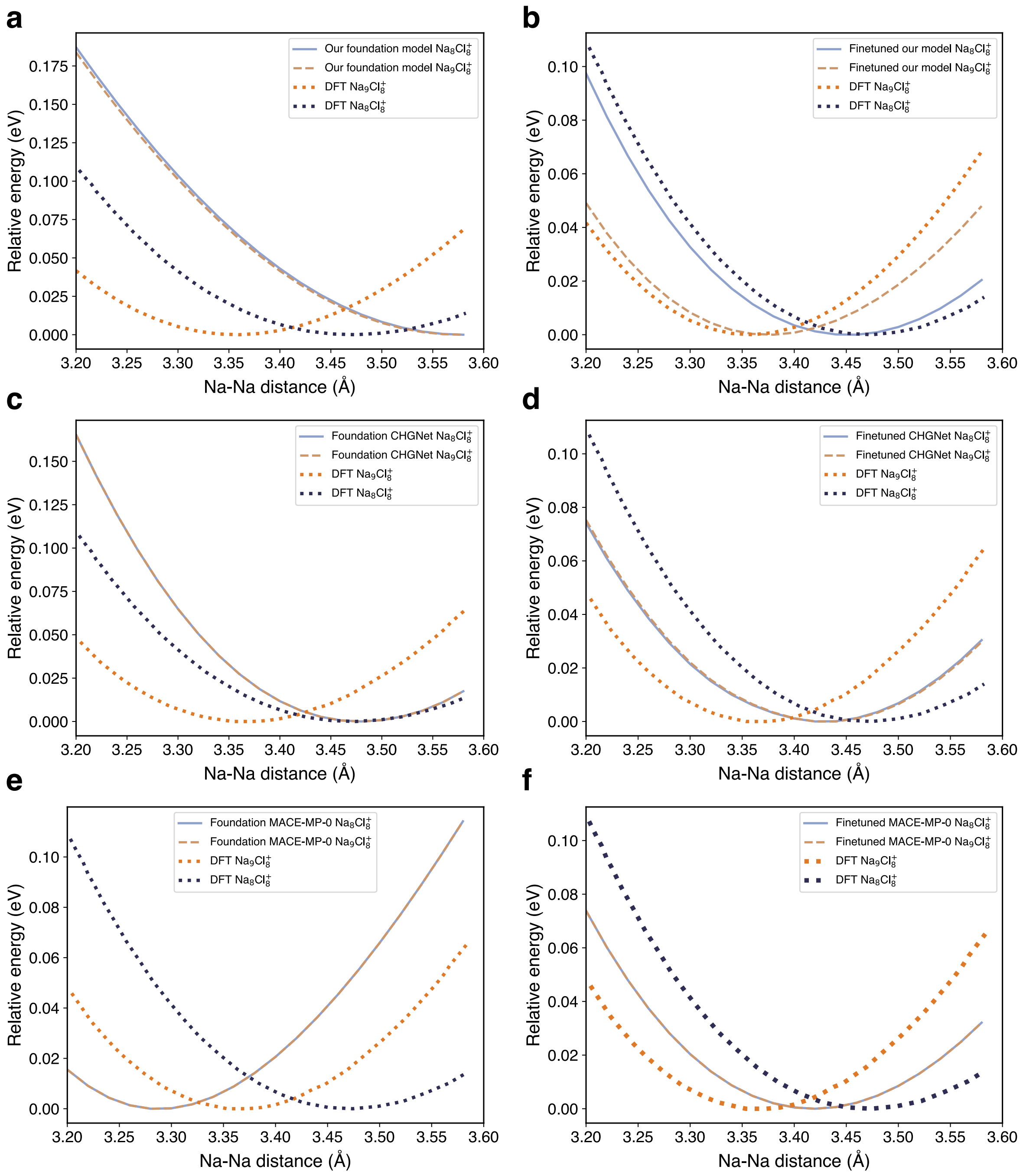


**Supplementary Fig. 12: Finetuning capacity of different foundation models for $Na_{8/9}Cl_8^+$ system.** The surfaces were generated by varying the position of the first Na atom relative to the second Na atom as described in Supplementary Fig. 1. Left column: foundation models' results of **a** our model, **c** CHGNet [6], and **e** MACE-MP-0 (large version) [10]. Right column: the finetuned models' results of **b** our model, **d** CHGNet, and **f** MACE-MP-0. Our model achieves good agreement with the density functional theory (DFT) reference, demonstrating its superior capability to refine the potential energy surface for complex charged systems.

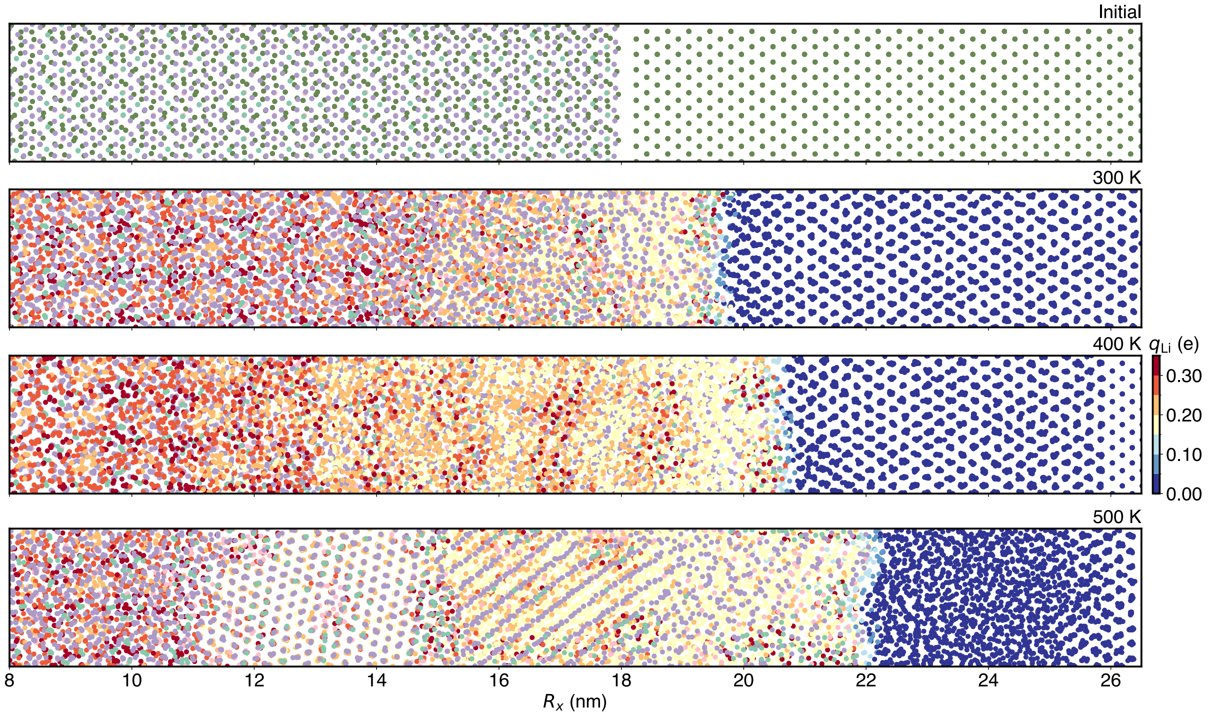


**Supplementary Fig. 13: Solid electrolyte interphase of the $Li_6PS_5Cl$/Li system.** A segment of the primary reaction zone, ranging from 8 to 26.5 nanometres along the *x* direction ($R_x$) is displayed. The elements are colour-coded for initial structures: Li in green, S in purple, P in pink, and Cl in light green. For final structures at 300 K, 400 K, and 500 K, the partial charges on lithium ($q_{\mathrm{Li}}$) ions are represented with colour coding to enhance the visibility of structural transformations during the formation of solid electrolyte interphase (SEI).

## Supplementary Tables 1-10

**Supplementary Table 1** Energy difference between wetting and non-wetting configurations for doped and pristine substrates ($E_{\text{wetting}} - E_{\text{non-wetting}}$) for density functional theory (DFT) [4], 4G-HDNNP [4], and our work, expressed in meV.

| | DFT [4] | 4G-HDNNP [4] | This work |
|---|---|---|---|
| Doped | −66.9 | −41.0 | −77.5 |
| Pristine | 934.8 | 975.0 | 930.5 |

**Supplementary Table 2** The mean absolute difference of density functional theory (DFT) Hirshfeld charges [28] and polarizable charge equilibration (PQEq) partial charges for the different charge state dataset, expressed in e.

| | $C_{10}H_2/C_{10}H_3^+$ | $Na_{8/9}Cl_8^+$ | $Ag_3^{+/-}$ | $Au_2$-MgO |
|---|---|---|---|---|
| Difference | 0.0182 | 0.396 | 0.210 | 0.0364 |

**Supplementary Table 3** The architectures for training different charge state dataset. $l_{\text{max}}$ is the maximum rotation order.

| Dataset | Settings | Maruf's NequIP [5] | This work |
|---|---|---|---|
| $C_{10}H_2/C_{10}H_3^+$ | Cutoff (Å) | 5.00 | 5.00 |
| | Number of layers | 8 | 8 |
| | $l_{\text{max}}$ | 1 | 1 |
| $Na_{8/9}Cl_8^+$ | Cutoff (Å) | N.A. | 5.29 |
| | Number of layers | N.A. | 5 |
| | $l_{\text{max}}$ | N.A. | 2 |
| $Ag_3^{+/-}$ | Cutoff (Å) | 5.29 | 5.29 |
| | Number of layers | 8 | 8 |
| | $l_{\text{max}}$ | 2 | 2 |
| $Au_2$-MgO | Cutoff (Å) | 5.50 | 5.50 |
| | Number of layers | 6 | 6 |
| | $l_{\text{max}}$ | 2 | 2 |
| BTA-Cu | Cutoff (Å) | 5.00 | 5.00 |
| | Number of layers | 6 | 6 |
| | $l_{\text{max}}$ | 1 | 1 |
| BTA ($H_2O$)-Cu | Cutoff (Å) | 5.00 | 5.00 |
| | Number of layers | 6 | 6 |
| | $l_{\text{max}}$ | 1 | 1 |

**Supplementary Table 4** Comparison of mean absolute errors in test set of our model and the model without polarizable long-range interactions (w/o-lr model).

| Model | Energy (meV·atom$^{-1}$) | Force (eV·Å$^{-1}$) | Stress (GPa) |
|---|---|---|---|
| Our model | 18 | 0.065 | 0.301 |
| w/o-lr model | 21 | 0.074 | 0.340 |

**Supplementary Table 5** Comparison of mean absolute errors in the test sets of the reference [11] of CHGNet [6], MACE-MP-0 (large version) [10], and our model.

| Test set | | CHGNet [6] | MACE-MP-0 [10] | Our model |
|---|---|---|---|---|
| MPtrj-random | Energy (meV·atom$^{-1}$) | 0.029 | 0.014 | 0.019 |
| | Force (eV·Å$^{-1}$) | 0.060 | 0.056 | 0.066 |
| | Stress (GPa) | 0.448 | 0.654 | 0.271 |
| Alexandria | Energy (meV·atom$^{-1}$) | 0.112 | 0.096 | 0.115 |
| | Force (eV·Å$^{-1}$) | 0.063 | 0.059 | 0.064 |
| | Stress (GPa) | 1.390 | 1.545 | 1.055 |
| MPF-Alkali-TP | Energy (meV·atom$^{-1}$) | 0.251 | 1.252 | 0.280 |
| | Force (eV·Å$^{-1}$) | 0.898 | 9.398 | 0.717 |
| | Stress (GPa) | 22.072 | 321.291 | 13.841 |
| MPF-TP | Energy (meV·atom$^{-1}$) | 0.241 | 292.408 | 0.521 |
| | Force (eV·Å$^{-1}$) | 1.388 | 1050.603 | 0.768 |
| | Stress (GPa) | 24.043 | 55355.387 | 15.445 |
| Random-TP | Energy (meV·atom$^{-1}$) | 0.518 | 8.946 | 3.338 |
| | Force (eV·Å$^{-1}$) | 2.048 | 47.509 | 1.119 |
| | Stress (GPa) | 20.696 | 2202.797 | 33.282 |

**Supplementary Table 6** The equilibrium bond lengths of $Au_2$ dimer absorbed on both Al-doped and undoped MgO surfaces.

| | | $Au_2$ on Al-doped MgO (Å) | $Au_2$ on MgO (Å) |
|---|---|---|---|
| True value of DFT | | 2.332 | 2.190 |
| Relative value compared to DFT | 4G-HDNNP [4] | +0.010 | −0.003 |
| | Bespoke model | −0.002 | 0.000 |
| | Our foundation model | −0.120 | +0.038 |
| | Foundation CHGNet [6] | −0.098 | +0.040 |
| | Foundation MACE-MP-0 [10] | +0.028 | +0.154 |
| | Finetuned our model | +0.002 | −0.002 |
| | Finetuned CHGNet | −0.002 | −0.004 |
| | Finetuned MACE-MP-0 | −0.068 | +0.034 |

**Supplementary Table 7** Final polarizable charge equilibration (PQEq) parameters used in the foundation model.

| Atom | $\chi^0$ (eV) | $\eta^0$ (eV) | $z$ | Radius (Å) | $K_s$ (kcal·mol$^{-1}$·Å$^{-2}$) |
|---|---|---|---|---|---|
| H | 4.72484 | 15.57338 | 1.00000 | 0.37100 | 2037.20061 |
| He | 9.66000 | 29.84000 | 1.00000 | 1.30000 | 1619.41057 |
| Li | 3.87172 | 14.85384 | 1.00000 | 0.69125 | 1083.03576 |
| Be | 4.87700 | 8.88600 | 1.00000 | 1.24000 | 59.29709 |
| B | 5.11000 | 9.50000 | 1.00000 | 0.82200 | 109.59198 |
| C | 5.34300 | 10.12600 | 1.00000 | 0.75900 | 198.84054 |
| N | 7.78778 | 10.80315 | 1.00000 | 0.71500 | 301.87609 |
| O | 8.30811 | 14.66128 | 1.00000 | 0.66900 | 414.04451 |
| F | 10.87400 | 14.94800 | 1.00000 | 0.70600 | 596.16463 |
| Ne | 11.04000 | 21.10000 | 1.00000 | 1.76800 | 842.11732 |
| Na | 2.84300 | 4.59200 | 1.00000 | 2.08500 | 13.77286 |
| Mg | 3.95100 | 7.38600 | 1.00000 | 1.50000 | 31.32676 |
| Al | 4.06000 | 7.18000 | 1.00000 | 1.20100 | 48.83290 |
| Si | 4.16800 | 6.97400 | 1.00000 | 1.17600 | 60.04769 |
| P | 6.52204 | 7.13703 | 1.00000 | 1.10200 | 91.47760 |
| S | 6.92800 | 8.97200 | 1.00000 | 1.04700 | 114.50472 |
| Cl | 8.56400 | 9.89200 | 1.00000 | 0.99400 | 152.32280 |
| Ar | 9.46500 | 12.71000 | 1.00000 | 2.10800 | 202.34215 |
| K | 2.42100 | 3.84000 | 1.00000 | 2.58600 | 7.71165 |
| Ca | 3.23100 | 5.76000 | 1.00000 | 2.00000 | 14.56420 |
| Sc | 3.39500 | 6.16000 | 1.00000 | 1.75000 | 18.65526 |
| Ti | 3.47000 | 6.76000 | 1.00000 | 1.60700 | 22.74409 |
| V | 3.65000 | 6.82000 | 1.00000 | 1.47000 | 26.77933 |
| Cr | 3.41500 | 7.73000 | 1.00000 | 1.40200 | 28.62618 |
| Mn | 3.32500 | 8.21000 | 1.00000 | 1.53300 | 35.32593 |
| Fe | 3.76000 | 8.28000 | 1.00000 | 1.39300 | 39.53139 |
| Co | 4.10500 | 8.35000 | 1.00000 | 1.40600 | 44.27516 |
| Ni | 4.46500 | 8.41000 | 1.00000 | 1.39800 | 48.83290 |
| Cu | 3.72900 | 5.00200 | 1.00000 | 1.43400 | 53.55866 |

| | | | | | |
|---|---|---|---|---|---|
| Zn | 5.10600 | 8.57000 | 1.00000 | 1.40000 | 57.75021 |
| Ga | 3.64100 | 6.32000 | 1.00000 | 1.21100 | 40.89454 |
| Ge | 4.05100 | 6.87600 | 1.00000 | 1.18900 | 56.86022 |
| As | 5.18800 | 7.61800 | 1.00000 | 1.20400 | 77.04494 |
| Se | 6.42800 | 8.26200 | 1.00000 | 1.22400 | 88.08056 |
| Br | 7.79000 | 8.85000 | 1.00000 | 1.14100 | 108.87334 |
| Kr | 8.50500 | 11.43000 | 1.00000 | 2.27000 | 133.65952 |
| Rb | 2.33100 | 3.69200 | 1.00000 | 2.77000 | 7.02929 |
| Sr | 3.02400 | 4.88000 | 1.00000 | 2.41500 | 12.03129 |
| Y | 3.83000 | 5.62000 | 1.00000 | 1.99800 | 14.62836 |
| Zr | 3.40000 | 7.10000 | 1.00000 | 1.75800 | 18.55104 |
| Nb | 3.55000 | 6.76000 | 1.00000 | 1.60300 | 21.15055 |
| Mo | 3.46500 | 7.51000 | 1.00000 | 1.53000 | 25.94248 |
| Tc | 3.29000 | 7.98000 | 1.00000 | 1.50000 | 29.12839 |
| Ru | 3.57500 | 8.03000 | 1.00000 | 1.50000 | 34.58997 |
| Rh | 3.97500 | 8.01000 | 1.00000 | 1.50900 | 38.61206 |
| Pd | 4.32000 | 8.00000 | 1.00000 | 1.54400 | 69.17994 |
| Ag | 4.43600 | 6.26800 | 1.00000 | 1.62200 | 48.97695 |
| Cd | 5.03400 | 7.91400 | 1.00000 | 1.60000 | 45.11735 |
| In | 3.50600 | 5.79200 | 1.00000 | 1.40400 | 32.55526 |
| Sn | 3.98700 | 6.24800 | 1.00000 | 1.35400 | 52.87639 |
| Sb | 4.89900 | 6.68400 | 1.00000 | 1.40400 | 50.31268 |
| Te | 5.81600 | 7.05200 | 1.00000 | 1.38000 | 60.37522 |
| I | 6.82200 | 7.52400 | 1.00000 | 1.33300 | 62.06798 |
| Xe | 7.59500 | 9.95000 | 1.00000 | 2.45900 | 82.11269 |
| Cs | 2.18300 | 3.42200 | 1.00000 | 2.98400 | 5.58842 |
| Ba | 2.81400 | 4.79200 | 1.00000 | 2.44200 | 8.36432 |
| La | 2.83550 | 5.48300 | 1.00000 | 2.07100 | 10.67729 |
| Ce | 2.77400 | 5.38400 | 1.00000 | 1.92500 | 11.21837 |
| Pr | 2.85800 | 5.12800 | 1.00000 | 2.00700 | 11.77531 |
| Nd | 2.86850 | 5.24100 | 1.00000 | 2.00700 | 10.57528 |
| Pm | 2.88100 | 5.34600 | 1.00000 | 2.00000 | 11.03202 |

| | | | | | |
|---|---|---|---|---|---|
| Sm | 2.91150 | 5.43900 | 1.00000 | 1.97800 | 11.52999 |
| Eu | 2.87850 | 5.57500 | 1.00000 | 2.22700 | 11.98786 |
| Gd | 3.16650 | 5.94900 | 1.00000 | 1.96800 | 14.13037 |
| Tb | 3.01800 | 5.66800 | 1.00000 | 1.95400 | 13.02211 |
| Dy | 3.05550 | 5.74300 | 1.00000 | 1.93400 | 13.55362 |
| Ho | 3.12700 | 5.78200 | 1.00000 | 1.92500 | 14.07050 |
| Er | 3.18650 | 5.82900 | 1.00000 | 1.91500 | 14.62836 |
| Tm | 3.25140 | 5.86580 | 1.00000 | 2.00000 | 15.23228 |
| Yb | 3.28890 | 5.93000 | 1.00000 | 2.15800 | 15.88822 |
| Lu | 2.96290 | 4.92580 | 1.00000 | 1.89600 | 15.16273 |
| Hf | 3.70000 | 6.80000 | 1.00000 | 1.75900 | 20.49776 |
| Ta | 5.10000 | 5.70000 | 1.00000 | 1.60500 | 25.34837 |
| W | 4.63000 | 6.62000 | 1.00000 | 1.53800 | 29.91565 |
| Re | 3.96000 | 7.84000 | 1.00000 | 1.60000 | 34.23337 |
| Os | 5.14000 | 7.26000 | 1.00000 | 1.70000 | 39.06632 |
| Ir | 5.00000 | 8.00000 | 1.00000 | 1.86600 | 43.69259 |
| Pt | 4.79000 | 8.86000 | 1.00000 | 1.55700 | 51.08672 |
| Au | 4.89400 | 5.17200 | 1.00000 | 1.61800 | 57.25236 |
| Hg | 6.27000 | 8.32000 | 1.00000 | 1.60000 | 66.14815 |
| Tl | 3.20000 | 5.80000 | 1.00000 | 1.53000 | 43.69259 |
| Pb | 3.90000 | 7.06000 | 1.00000 | 1.44400 | 47.57360 |
| Bi | 4.69000 | 7.48000 | 1.00000 | 1.51400 | 44.87347 |
| Po | 4.21000 | 8.42000 | 1.00000 | 1.48000 | 48.83290 |
| At | 4.75000 | 9.50000 | 1.00000 | 1.47000 | 55.34395 |
| Rn | 5.37000 | 10.74000 | 1.00000 | 2.20000 | 62.65353 |
| Fr | 2.00000 | 4.00000 | 1.00000 | 2.30000 | 6.83259 |
| Ra | 2.84300 | 4.86800 | 1.00000 | 2.20000 | 8.67007 |
| Ac | 2.83500 | 5.67000 | 1.00000 | 2.10800 | 10.34466 |
| Th | 3.17500 | 5.81000 | 1.00000 | 2.01800 | 10.34466 |
| Pa | 2.98500 | 5.81000 | 1.00000 | 1.80000 | 13.07337 |
| U | 3.34100 | 5.70600 | 1.00000 | 1.71300 | 13.33589 |
| Np | 3.54900 | 5.43400 | 1.00000 | 1.80000 | 13.38967 |

| | | | | | |
|---|---|---|---|---|---|
| Pu | 3.24300 | 5.63800 | 1.00000 | 1.84000 | 13.55362 |
| Am | 2.98950 | 6.00700 | 1.00000 | 1.94200 | 14.25166 |
| Cm | 2.83150 | 6.37900 | 1.00000 | 1.90000 | 14.43755 |
| Bk | 3.19350 | 6.07100 | 1.00000 | 1.90000 | 14.62836 |
| Cf | 3.19700 | 6.20200 | 1.00000 | 1.90000 | 16.19823 |
| Es | 3.33300 | 6.17800 | 1.00000 | 1.90000 | 16.85603 |
| Fm | 3.40000 | 6.20000 | 1.00000 | 1.90000 | 13.95226 |
| Md | 3.47000 | 6.22000 | 1.00000 | 1.90000 | 18.24526 |
| No | 3.47500 | 6.35000 | 1.00000 | 1.90000 | 20.24779 |

**Supplementary Table 8** The optimized polarizable charge equilibration (PQEq) Ag parameters for $Ag_3^{+/-}$ cluster.

| Atom | $\chi^0$ (eV) | $\eta^0$ (eV) | $z$ | Radius (Å) | $K_s$ (kcal·mol$^{-1}$·Å$^{-2}$) |
|---|---|---|---|---|---|
| Ag | 3.50747 | 4.80698 | 1.00000 | 1.17602 | 221.26430 |

**Supplementary Table 9** Comparison of our bespoke models and cace-lr models [25] in Ko et al. [4] datasets.

| Dataset | | cace-lr | This work |
|---|---|---|---|
| $C_{10}H_2/C_{10}H_3^+$ | Energy (meV/atom) | 0.73 | 0.44 |
| | Force (eV/Å) | 0.037 | 0.023 |
| $Na_{8/9}Cl_8^+$ | Energy (meV/atom) | 0.21 | 0.16 |
| | Force (eV/Å) | 0.010 | 0.005 |
| $Ag_3^{+/-}$ | Energy (meV/atom) | 0.162 | 4.87 |
| | Force (eV/Å) | 0.029 | 0.028 |
| $Au_2$-MgO | Energy (meV/atom) | 0.073 | 0.13 |
| | Force (eV/Å) | 0.008 | 0.006 |

**Supplementary Table 10** Final polarizable charge equilibration (PQEq) parameters used for Ag and Au in the bespoke models for Supplementary Note 11.

| Atom | $\chi^0$ (eV) | $\eta^0$ (eV) | $z$ | Radius (Å) | $K_s$ (kcal·mol$^{-1}$·Å$^{-2}$) |
|---|---|---|---|---|---|
| Ag | 3.50747 | 4.80698 | 1.00000 | 1.17602 | 221.26430 |
| Au | 5.76708 | 6.91695 | 1.00000 | 0.55600 | 740.00000 |

## Supplementary References


1. Naserifar, S., Brooks, D. J., Goddard, W. A. & Cvicek, V. polarizable charge equilibration model for predicting accurate electrostatic interactions in molecules and solids. *J. Chem. Phys.* **146**, 124117 (2021).
2. Oppenheim, J.J., Naserifar, S. & Goddard, W. A. Extension of the polarizable charge equilibration model to higher oxidation states with applications to Ge, As, Se, Br, Sn, Sb, Te, I, Pb, Bi, Po, and At elements. *J. Phys. Chem. A* **122**, 639–645 (2018).
3. Kwon, S., Naserifar, S., Lee, H. M. & Goddard, W. A. polarizable charge equilibration model for transition-metal elements. *J. Phys. Chem. A* **122**, 9350–9358 (2018).
4. Ko, T. W., Finkler, J. A., Goedecker, S. & Behler, J. A fourth-generation high-dimensional neural network potential with accurate electrostatics including non-local charge transfer. *Nat. Commun.* **12**, 398 (2021).
5. Maruf, M. U., Kim, S. & Ahmad, Z. Equivariant machine learning interatomic potentials with global charge redistribution. Preprint at https://arxiv.org/abs/2503.17949 (2025)
6. Deng, B. et al. CHGNet as a pretrained universal neural network potential for charge-informed atomistic modelling. *Nat. Machine Intell.* **5**, 1031–1041 (2023).
7. Jain, A. et al. The Materials Project: a materials genome approach to accelerating materials innovation. *APL Mater.* **1**, 011002 (2013).
8. Delacourt, C., Poizot, P., Tarascon, J. M. & Masquelier, C. The existence of a temperature-driven solid solution in $Li_xFePO_4$ for $0 \leq x \leq 1$. *Nat. Mater.* **4**, 254–260 (2005).
9. Yang, J. & Tse, J. S. Li ion diffusion mechanisms in $LiFePO_4$: an *ab initio* molecular dynamics study. *J. Phys. Chem. A* **115**, 13045–13049 (2011).
10. Batatia, I. et al. A foundation model for atomistic materials chemistry. Preprint at https://arxiv.org/abs/2401.00096 (2024).
11. Yang, H. et al. MatterSim: A deep learning atomistic model across elements, temperatures and pressures. Preprint at https://arxiv.org/abs/2405.04967 (2024).
12. Schmidt, J. et al. Machine-learning-assisted determination of the global zero-temperature phase diagram of materials. *Adv. Mater.* **35**, 2210788 (2023).
13. Chen, C. & Ong, S. P. A universal graph deep learning interatomic potential for the periodic table. *Nat. Comput. Sci.* **2**, 718–728 (2022).
14. Koski, J.P. et al. Water in an external electric field: comparing charge distribution methods using ReaxFF simulations. *J. Chem. Theory Comput.* **18**, 580–594 (2022).
15. Nosé, S. A unified formulation of the constant temperature molecular dynamics methods. *J. Chem. Phys.* **81**, 511–519 (1984).
16. Hoover, W. G. Canonical dynamics: equilibrium phase–space distributions. *Phys. Rev. A Gen. Phys.* **31**, 1695–1697 (1985).
17. Perdew, J. P., Burke, K. & Ernzerhof, M. Generalized gradient approximation made simple. *Phys. Rev. Lett.* **77**, 3865 (1996).
18. Zhang, Y. & Jiang, B. Universal machine learning for the response of atomistic systems to external fields. *Nat. Commun.* **14**, 6424 (2023).
19. Yang, Y. *et al.* Quantum mechanical static dipole polarizabilities in the QM7b and AlphaML showcase databases. *Sci. Data* **6**, 152 (2019).
20. Wilkins, D. M. et al. Accurate molecular polarizabilities with coupled cluster theory and machine learning. *Proc. Natl. Acad. Sci. USA* **116**, 3401–3406 (2019).
21. Blum, L. C. & Reymond, J.-L. 970 million druglike small molecules for virtual screening in the chemical universe database gdb-13. *J. Am. Chem. Soc.* **131**, 8732–8733 (2009).
22. Montavon, G. et al. Machine learning of molecular electronic properties in chemical compound space. *New J. Phys.* **15**, 095003 (2013).

23. Liu, K. et al. Shift-collapse acceleration of generalized polarizable reactive molecular dynamics for machine learning-assisted computational synthesis of layered materials. In *2018 IEEE/ACM 9th Workshop on Latest Advances in Scalable Algorithms for Large-Scale Systems* (scalA, 2018)
24. Shannon, R. D. Revised effective ionic-radii and systematic studies of interatomic distances in halides and chalogenids. *Acta Crystallogr. A* **32**, 751–767 (1976).
25. Kim, D., King, D.S., Zhong, P. & Cheng, B. Learning charges and long-range interactions from energies and forces. Preprint at https://arxiv.org/abs/2412.15455 (2024)
26. Ghosez, P., Gonze, X., Lambin, P. & Michenaud, J.-P. Born effective charges of barium titanate: Band-by-band decomposition and sensitivity to structural features. *Phys. Rev. B* **51**, 6765–6768 (1995).
27. Das, T., Merinov, B. V., Yang, M. Y. & Goddard, W. A. Structural, dynamic, and diffusion properties of a $Li_6(PS)_4Cl$ superionic conductor from molecular dynamics simulations; prediction of a dramatically improved conductor. *J. Mater. Chem. A* **10**, 16319–16327 (2022).
28. Hirshfeld, F. L. Bonded-atom fragments for describing molecular charge densities. *Theor. Chim. Acta* **44**, 129–138 (1977).
